# An Objective Measure of Noseband Tightness and Its Measurement Using a Novel Digital Tightness Gauge

**DOI:** 10.1371/journal.pone.0168996

**Published:** 2017-01-03

**Authors:** Orla Doherty, Thomas Conway, Richard Conway, Gerard Murray, Vincent Casey

**Affiliations:** 1 Department of Life Sciences, University of Limerick, Limerick, Ireland; 2 Department of Electronic and Computer Engineering, University of Limerick, Limerick, Ireland; 3 Aaron Value Adding Services Ltd.,Unit M7, Smithstown Industrial Estate, Shannon, Co.Clare, Ireland; 4 Department of Physics,University of Limerick, Limerick, Ireland; University of Minnesota, UNITED STATES

## Abstract

Noseband tightness is difficult to assess in horses participating in equestrian sports such as dressage, show jumping and three-day-eventing. There is growing concern that nosebands are commonly tightened to such an extent as to restrict normal equine behaviour and possibly cause injury. In the absence of a clear agreed definition of noseband tightness, a simple model of the equine nose-noseband interface environment was developed in order to guide further studies in this area. The normal force component of the noseband tensile force was identified as the key contributor to sub-noseband tissue compression. The model was used to inform the design of a digital tightness gauge which could reliably measure the normal force component of the noseband tensile force. A digital tightness gauge was developed to measure this parameter under nosebands fitted to bridled horses. Results are presented for field tests using two prototype designs. Prototype version three was used in field trial 1 (n = 15, frontal nasal plane sub-noseband site). Results of this trial were used to develop an ergonomically designed prototype, version 4, which was tested in a second field trial (n = 12, frontal nasal plane and lateral sub-noseband site). Nosebands were set to three tightness settings in each trial as judged by a single rater using an International Society for Equitation Science (ISES) taper gauge. Normal forces in the range 7–95 N were recorded at the frontal nasal plane while a lower range 1–28 N was found at the lateral site for the taper gauge range used in the trials. The digital tightness gauge was found to be simple to use, reliable, and safe and its use did not agitate the animals in any discernable way. A simple six point tightness scale is suggested to aid regulation implementation and the control of noseband tightness using normal force measurement as the objective tightness discriminant.

## Introduction

Studies of the effects of force induced pressure on human tissue [1] are particularly important in medicine and biomedical engineering where they have lead to significant advances in surgical [2] and emergency care [3, 4]. Externally applied pressure comes into play in many veterinary and equestrian activities [5], wound treatment being an obvious example. Pressure is inadvertently or purposefully applied in the use and control of the horse [6, 7] and so is significant in equitation science. Despite this, there are very significant gaps in our knowledge and understanding of the effects of externally applied forces, sustained or transient, and pressures in uniform or gradient form, on animal tissue and animal behaviour. Welfare concerns raised in relation to restrictive bridle nosebands [8] have stimulated interest specifically in the noseband-nose environment [9, 10] and in the development of measurement techniques [11, 12] which could provide a basis for improved understanding. There has long been a recognition of the need to regulate the use of restrictive nosebands and indeed rules and guidelines have been developed [13–15]. Regulation is generally fairest and most successful where an accessible objective measure exists. Noseband tightness has been muted as a relevant measure in relation to restrictive nosebands but there is no clear agreed definition of tightness in this context. A count of the number of fingers (human hand) which may be inserted between the band and the tissue [13], while useful in much the same way as measuring an animal’s height using human hand width, does not meet basic criterion in relation to scientific measurement and therefore is inadequate as a basis for robust regulation. Noseband tension (force per unit width of noseband) is a possible contender particularly if rein tension technology could be adapted to the noseband environment. However, the relative complexity of the noseband-nose interface compared to the simple ‘free-standing’ rein environment prompts a closer examination of the former application environment.

## Background

### Curvature Model of Equine Nose-Noseband Interface

The primary mechanical action produced in a tight noseband when a handler fastens it on a horse is to produce a tensile or tensional force along the length of the band. The noseband will generally have some freedom to rotate around the nose and so this tensional force will tend to equalise around the band and settle at a constant value throughout its length. However, because the band curves around prominent support tissue, the constant tensile force will give rise to a non-zero ‘normal force’ component directed towards the tissue, effectively compressing it. The magnitude of this normal force component will depend on the magnitude of the tensile force but crucially will also depend on the local tissue curvature. The equine nose is not a regular geometric shape such as a cylinder or sphere (with constant curvature) and so the effect of a constant tensile force on nose tissue will vary from place to place under the noseband, as the curvature of the nose varies. A comprehensive model of the noseband-nose interface for tight nosebands needs to take noseband tension, noseband profile and sub-noseband surface topology into account in order to provide a realistic map of the ‘force-print’ of a tight noseband on nose tissue. However, it is possible to gain some useful insights on noseband-tissue mechanical interactions using a greatly simplified curvature model of the equine nose-noseband environment.

The following assumptions and simplifications are made in order to develop a simple one dimensional static model of the equine nose-noseband environment from a measurement perspective. **1.** The tensional force (stretching force) in the tightened noseband is assumed constant along the length of the noseband. This is reasonable provided the noseband is free to slip/slide circumferentially over the nose to relieve localised force concentrations. **2.** The noseband length is much greater than its width, i.e. on the order of ten times its width. **3.** Curvature (changes of shape) of the equine nose across the width of the noseband, i.e. longitudinal tapering, will be small relative to the curvature along its length, i.e. circumferentially. As a consequence of **2.** and **3.** noseband deformation/curvature will occur primarily along its length as it encircles the nose. **4.** The nose will deform to accommodate the tightened noseband but the degree of accommodation possible will be limited due to hard tissue, i.e. bony inclusions. **5.** The horse does not chew or otherwise change the configuration of its nose under the noseband during a measurement, i.e. the model is limited to static rather than dynamic analysis. **6.** The profile of the inner surface of the noseband is flat. **7.** The noseband strap is non-elastic, i.e. does not stretch appreciably under normal use conditions.

**Fig 1:**
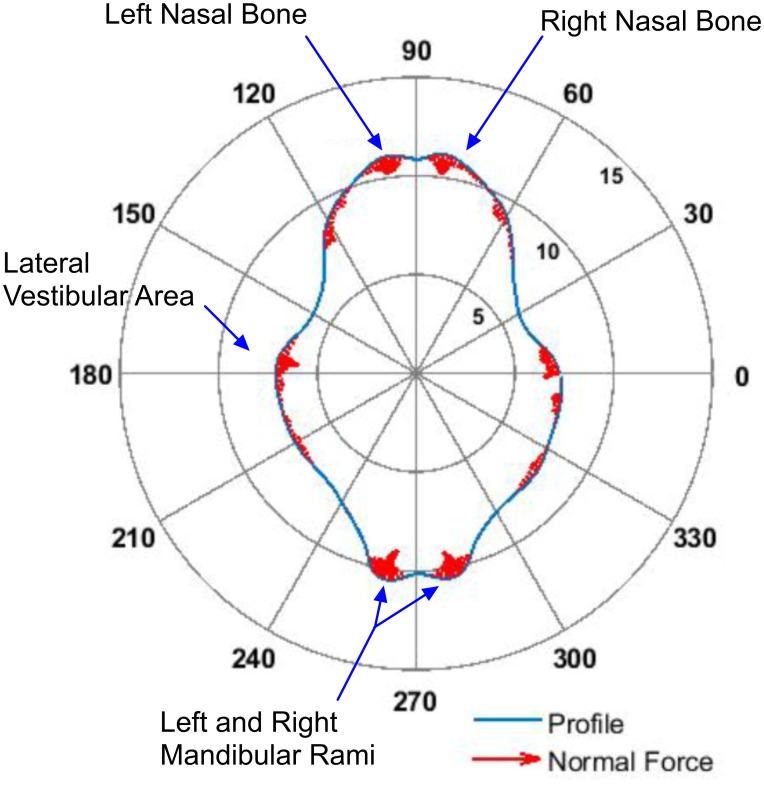
Polar (radial distance, cm, and angle, degrees) plot of nose section profile (blue line) and normal force vector scaled to local curvature (red arrows). The normal force vector is scaled to zero for negative or zero curvature areas.

A tight noseband curves around the nose and encircles it. Based on the assumptions listed above, the curvature *κ* is simply 1/*R* where *R* is the local radius of curvature or radius of the osculating circle [16]. This is an imaginary circle formed by completing the arc defined by the tissue surface locally. Only regions of positive curvature (radius of curvature directed in and through the tissue) need to be considered since the noseband will not track regions of negative tissue curvature, i.e. does not conform to the tissue in such regions. Highly curved regions (small *R*) will be subject to higher normal (compressive) forces than less curved regions (large *R*) for a given applied tensile force. This is expressed in LaPlace’s law [17] where the sub-noseband pressure *P* is determined by *P* = *κT*/*W* where *T* is the tensile force in the noseband and *W* is the noseband width. Alternatively, the tensile force at any point may be resolved into two components, a tangential component and a normal (tissue directed) component which produces the pressure in the support tissue. LaPlace’s law takes the form,

N=u×T×κ (1)

where *u* is a constant determined by the local geometry and units system chosen. Therefore, a mapping of the curvature around a typical equine nose section at the noseband level should identify anatomical sites with large positive curvature which are likely to experience relatively large normal force components (and pressures) due to the use of restrictive nosebands. The circumferential profile of the nose at the noseband is required in order to calculate the curvature at each point along the noseband. Profile sections may be obtained experimentally using flexible rulers [11] or using inexpensive profilometers (see S1 Fig). However, it is difficult to obtain a complete profile using such techniques. In an attempt to examine an entire nose section, an equine head section provided online by the Online Veterinary Anatomy Museum(OVAM) [18] was scaled to the dimensions of a living adult horse (S2 Fig) and used to generate a curvature map for the entire section, Fig 1, using a custom MATLAB (R2015b, Mathworks, Natick, Massachusetts, U.S.A.) script, see S1 File.

**Fig 2:**
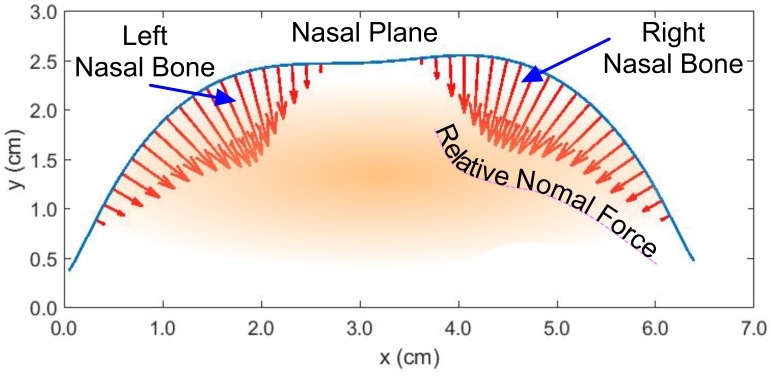
Relative normal force component over nasal bone regions for a constant tension noseband.

Tissue regions of high positive curvature will experience relatively large normal forces. Soft tissue areas such as muscle and mucus membranes covering the premolar teeth will deform effectively expanding the area of contact between the noseband and the tissue redistributing the normal force to some extent as the tightened noseband is accommodated. Ultimately, the degree of accommodation possible will be limited by hard tissue inclusions such as the teeth in the case of the lateral aspect of the face. Areas where the curvature is large but which have only a thin soft tissue overlayer, i.e. little accommodation, will experience the largest normal forces. This is the situation for the nasal bones and mandibular rami as indicated by the red regions of the profile. Regions where the curvature is negative, such as the inter nasal bone and inter mandibular rami areas, will experience zero normal force as the noseband spans linearly over them in a process often referred to as ‘hammocking’ [19]. An enlarged view of the relative variation in this normal force component, based on this simple curvature model, is shown in Fig 2 for the frontal nasal plane. The force is concentrated, as one would expect, at the frontal nasal bone regions. The actual force will scale with noseband tensile force according to Eq 1.

The situation for real nosebands is complicated by the fact that the noseband may not necessarily present a flat inner surface across its width but may itself be curved (contrary to assumption 6 above). The model developed here neglects this and the tapering of the equestrian nose (assumption 3) which, together, demand a 2D curvature model. Normal force estimates from this 1D model will therefore be conservative and are likely to err on the low side [11]. In addition, foam padding may be used under all or some of the noseband. This will provide some degree of redistribution of the forces coupled to the nose and may also impact the band curvature but such effects have not been considered in the model.

To summarise, the actual force applied by a noseband to underlying support tissue is determined by both the noseband tensile force and the local tissue curvature. The curvature model of the equine noseband-nose interface identifies the nasal plane (nasal bones), super-premolar cheek region and the mandibular rami as sites likely to experience relatively large normal forces due to restrictive nosebands and therefore are areas of primary interest as candidate noseband tightness measurement sites.

### Noseband Tightness Gauge Design Requirements

In addition to the normal requirements for a measurement instrument such as accuracy, reliability and reproducibility, there are additional application environment specific design characteristics for a noseband tightness gauge. Competition officials and stewards routinely carry out multiple checks on a wide range of animal-rider combinations at multiple events at a given venue. A noseband tightness gauge, if it is to be used in these checks, must be portable, easy to carry on the person and easy to operate in one hand. It should be battery operated and have a low power requirement in order to extend the recharge interval. The gauge should be minimally invasive in the sense that it should not be necessary to adjust the noseband or other elements of the bridle in order to take a measurement. All measurement involves some degree of interference or disturbance of the measurement environment. Gauge design must minimise this so that the measurement reflects as close as possible the true use condition. When deployed, the gauge must be safe and not pose an injury risk to the horse, rider, handler or steward. Furthermore, it should not pose an infection transmission risk, i.e. should be sterile or easily cleaned between animals. Use of the device must not frighten or disturb the animal. Since the noseband inspection is likely to be one of many specific checks, the tightness gauge must provide a stable, easily interpreted tightness indicator using a simple measurement protocol which seamlessly integrates with existing inspection protocols. Finally, the cornerstone of scientific measurement is traceability. In this context, this means that it must be possible to calibrate the instrument against physical standards appropriate to the specific measurement. For a force based gauge this will be a certified weight or set of weights traceable to national and international authority standards.

### Noseband tightness and its measurement

A large variety of small footprint electrical, electronic and microelectromechanical sensors and transducers exist for force and pressure measurement which may be adapted to a wide range of measurement environments. The living tissue-leather interface environment presents special challenges for these technologies which are developed specifically for fluids (gases and liquids) in the case of pressure sensors [19] and rigid elements in the case of force sensors. Tissue and leather do not fit into a simple physical phase classification category and so special housings and couplers, specific to the actual interface of interest, must be devised if the sensor technology is to be deployed directly at the measurement site. While the value of the actual normal force at the prominent nasal bone and mandibular rami areas is of particular interest when assessing the effect of tight nosebands, the noseband will be at its tightest at these sites and so inserting a probe at such sites would risk adding appreciably to the local tissue compression and so is excluded on animal safety/welfare grounds. The mandibular rami site is less than ideal for measurement as it is often covered by a noseband cushioning pad, unrepresentative of the rest of the noseband. Both the cheek plane and frontal nasal plane (internasal bone region) are relatively accessible. However, the preferred choice of measurement site is the frontal nasal plane on account of the clear landmarks available for this site and because it is of well defined form, i.e. has a clear stable geometry.

Mechanically, noseband tensile force is the primary force acting in the nose-noseband environment. However, as is clear from the simple 1D model developed above, the local tissue curvature determines the degree, if any, to which this tensile force couples to the underlying tissue via the variable normal force component. Thus while tension might be the obvious parameter choice for noseband tightness, particularly since bridle rein tension technology is already well established in equitation science [20–22], it is the normal force component of this tension which acts on the underlying tissue and is therefore of more interest from a noseband-nose interaction perspective. It is also worth noting that rein tension gauges are generally linked into the rein or cheek piece, i.e. connected in series with it, and even if current technology were scaled down to dimensions suitable for nosebands, the necessary stringent safety requirements are likely to make this approach impractical. However, an interesting approach which could avoid these risks was tested recently [23] in a research setting.

**Fig 3:**
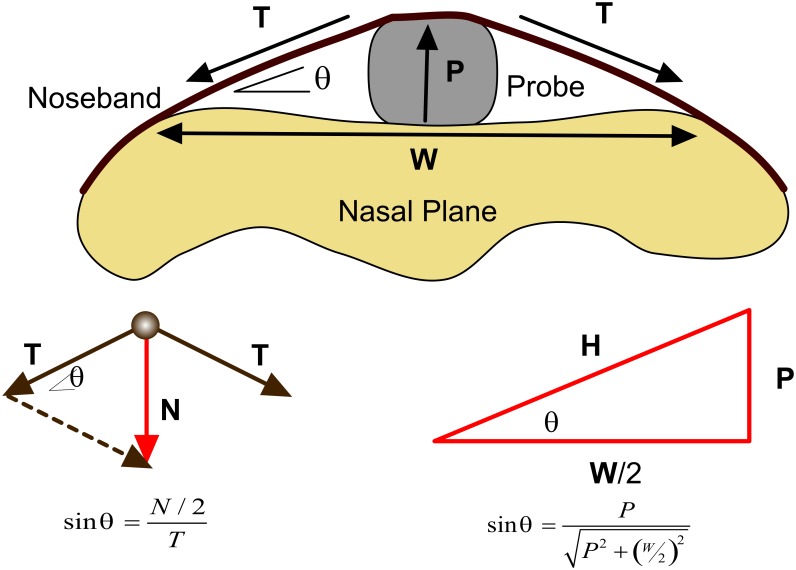
Schematic representation of the probe inserted between the noseband and midline of the nasal plane. *N* is the noseband force, *L* is the measured force/load, *P* is the height of the probe and *W* is the lift-off width at the nasal plane.

The concept of ‘finger’ based testing of tack band tightness is well established in equitation [6]. For this reason, and to avoid measurement artifacts which commonly arise with direct sensor (force and pressure) deployment, a lift-off measurement principle based on an insertable finger probe was identified as the most appropriate transduction technique for the application need. The finger probe couples directly to the local normal component of the noseband tensile force, while at the same time linking to remote environmentally isolated and protected sensors [24]. A better understanding of the finger probe measurement principle may be gained by reference to a specific measurement site on a horse such as the nasal plane illustrated schematically in Fig 3. The finger probe is necessarily intrusive, i.e. it lifts the noseband away from the tissue to a height, *P* at the measurement site in order to ‘sense’ the normal component of tension. Clearly, this lifting will tighten the noseband further (through effective contraction) and so it is necessary to be able to estimate the actual effective contraction due to lifting, to ascertain its impact. The effective contraction in noseband length, Δ*C*, due to a lift-off height *P* is given as a function of nasal plane width *W* by,

$$\Delta C=2\sqrt{P^{2}+{W/2}^{2}}-W$$ (2)

neglecting the slight concavity of the nasal bone. This relationship may be used to calculate the effective contraction length directly using appropriate measurement data or alternatively may be read from plots such as the one shown in S3 Fig for a specific configuration.

The lift-off finger probe measures the normal component *N* of the noseband tensile force *T*. Comparison of the free body force diagram geometry and the lift-off geometry, Fig 3, provides a relationship between *N* and *T* for the nasal plane application environment geometry,

$$T=\frac{1}{2}\frac{\sqrt{P^{2}+{\left(W/2\right)}^{2}}}{P}N$$ (3)

which may be simplified to *T* = *MN* where *M* is a multiplier factor given by,

$$M=\frac{1}{2}\frac{\sqrt{P^{2}+{\left(W/2\right)}^{2}}}{P}$$ (4)

when *W* is also measured. This allows one estimate the noseband tensile force from the measured normal component for the various anatomical geometries likely to arise or likely to be of interest in equitation.

### Design Implementation

**Fig 4:**
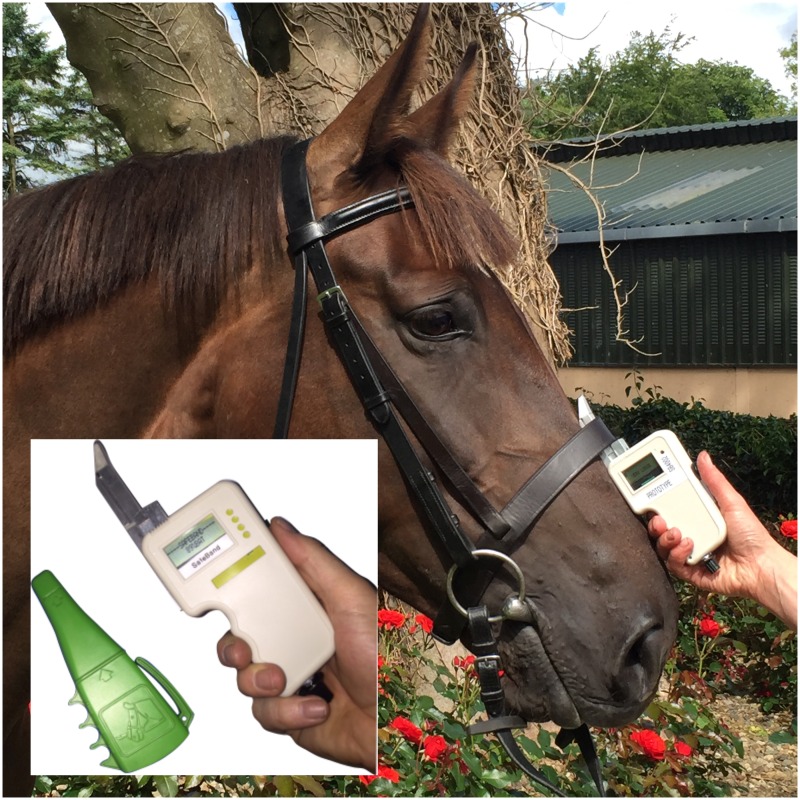
Digital tightness gauge, version 4 (DTGv4) deployed at a frontal site—nasal plane. Inset shows a close up of the gauge along side the ISES taper gauge.

A standard industry test finger is typically in the range 12–18 mm diameter. The ISES taper gauge varies in height from 10 mm at the tip to 16 mm at the base (corresponding width ranging from 16–38 mm tip to base). For this application, a simple square finger probe geometry of height/width (15 mm) was chosen largely as a compromise between the dimensions given above and to ensure ease of measurement, Fig 4. The finger has an overall length of approximately 55 mm comprising a tapered tip (20 mm) to facilitate lifting of the noseband and insertion of the measurement section of the finger (35 mm). Details of the finger probe and measurement system are revealed in a recent patent application [24]. The finger probe is inserted under the noseband at the site of interest. The digital tightness gauge provides a direct estimate of the normal force applied by the noseband to the underlying tissue. In-built firmware maps the measured load to a simple six-point tightness scale for display on an integrated backlight display, see Table 1.

**Table 1:** Digital tightness gauge, tightness scale.

Scale	Normal Force (N)
*VeryLoose*	0 to 5
*Loose*	6 to 10
*ModeratelyTight*	11 to 20
*Tight*	21 to 40
*VeryTight*	41 to 60
*ExtremelyTight*	Greater Than 60

The contraction length is estimated at less than 1 cm for a probe height of 15 mm and for nasal plane widths in the range 4–8 cm, see Eq 2. Concavity of the nasal plane along the midline as well as some degree of tissue accommodation will tend to reduce the effective lift-off height *P* below the nominal value further reducing the contraction effect. This contraction is likely to correspond to additional tightening of the noseband by half to two thirds of a normal noseband punch hole spacing.

**Fig 5:**
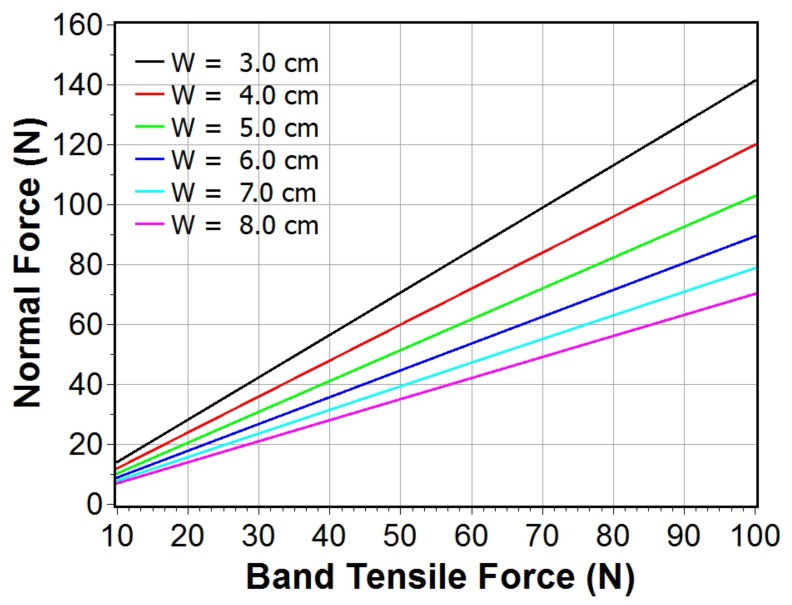
Relationship between measured normal force and band tensile force for various lift off widths.

It is clear from Eq 4 that the digital tightness gauge measured force will scale with the noseband tensile force. For a probe height of 15 mm the scale/multiplier factor is close to unity for lift-off widths in the range 4–6 cm which, in the absence of actual population metrics, was deemed to correspond approximately to a nasal plane width range likely to arise in an adult horse population. At measurement sites such as the cheek, i.e. lateral locations, where the lift-off width may be larger, the digital tightness gauge measured normal force will be significantly lower than the band tensile force, reflecting the generally lower tissue curvatures in this area. Therefore, while the finger probe dimensions are tailored specifically to the nasal plane measurement site where the application environment is reasonably well defined, it may be used for other sites, particularly if the noseband is so tight that the nasal plane site is inaccessible. However, an additional lift off width measurement would be required in order to estimate the actual tension in the band by reference to Fig 5 or using Eq 3 directly. In either case, the digital tightness gauge should prove useful for like-for-like comparisons of noseband tightness within competition classes and between individual horses since the probe height is constant. Additionally, with relevant expert group input, it should be sufficient to allow the development of a simple tightness scale to help formulate guidelines regarding permitted or acceptable levels of noseband tightness.

A bluetooth wireless module was included in the digital tightness gauge to facilitate data streaming for development purposes. A key feature of the design is that the gauge is largely independent of noseband loading point on the finger probe [24]. Details and results of device field trials are presented below.

## Materials and Methods

The protocol and conduct of these studies were approved by the University of Limerick Animal Ethics Committee: 2016-1-1-ULAEC and 2016-1-2-ULAEC.

### Digital Tightness Gauge Field Trials

Two field trials were carried out at a private breeding and training yard. Version three of the digital tightness gauge, DTGv3, was used for the first field trial while an ergonomically redesigned gauge, DGTv4, see Fig 4, was used for the second trial. All horses were either in training or had been previously used for riding but were retired for breeding purposes. Snaffle bridles with cavesson nosebands were used and horses were restrained by familiar handlers. All bridles were fitted with either a single-jointed full cheek snaffle bit or a single-jointed Baucher snaffle bit. A habituation period was allowed before the introduction of either the ISES taper gauge or the digital tightness gauge probe beneath the noseband, by holding the gauge or probe on the surface of the noseband, at the frontal nasal plane for a minimum of 3 seconds (or a longer duration if deemed necessary based on the behaviour of the horse). The width of each noseband was measured using a callipers designed to measure skin sickness in the tuberculosis skin test. Food was withheld from each horse for a minimum 10 minute period prior to data collection to minimise chewing or other oral behaviours during data collection. The ISES taper gauge was used to assist in assessing and adjusting the noseband tightness. Two sites, a frontal site and a lateral site, were used between the two studies. The frontal site corresponded to the region under the noseband between the rostral margin of the facial crest and the caudal margin of the noseband. The lateral site corresponded approximately to the area where the lateral nasal artery and vein pass under the noseband. Three taper gauge tightness levels were used on each horse. Measurements at these sites are represented by a four letter code where the first letter identifies either a frontal (F) or lateral (L) site and the remaining three digits identify the taper gauge tightness: two fingers 2F0; one finger 1F0; half finger 0F5.

The fifteen horses used in trial one ranged in age from four to twelve years. All bridles were initially fitted with an opened noseband before being adjusted to the taper gauge setting specified by the random order sequence for the study. The DTGv3 probe was inserted under the noseband at the frontal nasal plane and aligned with the rostral midline. The lateral noseband site was not used in this trial. The noseband applied force indicated on the instrument was recorded once oral immobility had been established. Where mouth movements were displayed, readings were recorded once a continuous mouth immobility period of at least three seconds had occurred and continued for the duration of the reading. The bridle was left in place but the noseband was opened and left loose once a reading had been logged. The procedure was repeated for the other two ISES taper gauge settings allowing at least a thirty minute rest period between each bridle noseband tightness reading. All horses were individually stabled. The horses were divided into two groups (a group of seven and a group of eight) and the study was completed in the first group before proceeding to the second group. The trial was completed in a single day over a four hour test period.

Twelve horses, four of whom had been used in trial one, ranging in age from four to twelve years, were used in the second trial. The horses were divided into two groups, a first group of four and a second group of eight. A stable arrangement of eight stables, four on either side of an access corridor (American barn-style building) was used for the data logging. Each group of horses was taken from their individual stables and placed in the ‘data-logging’ stables with two stables either side of the corridor. Both frontal and lateral noseband sites were used in this trial. The noseband was initially left unfastened. Following a procedure similar to study one, the ISES taper gauge was used to adjust the noseband tightness to one of the three prescribed tightness settings: 2F0; 1F0; 0F5. DTGv4 was then used to measure the noseband applied normal force at both the frontal and lateral locations. Each horse was given a rest period of at least thirty minutes, with the bridle removed, between readings at the different tightness settings. All data was collected on the same day over a nine hour period.

## Results

### Gauge Calibration

**Fig 6:**
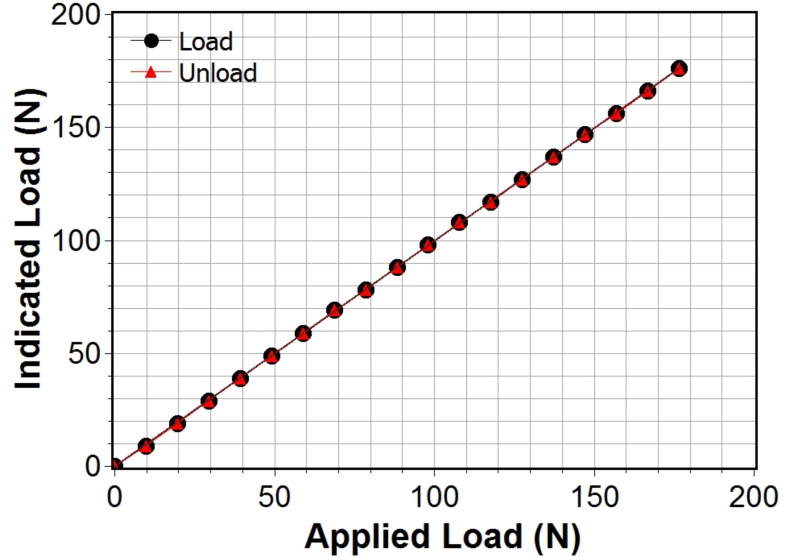
Digital tightness gauge indicated load for loading/unloading using dead weights in the range 0–20 kg, i.e. 0–196 N.

The digital tightness gauges were calibrated before commencement of each field trial and calibration was rechecked after field trial completion. The gauge uses a two-point calibration procedure whereby the operator is prompted to apply zero load followed by a 10 kg load to the finger probe. A calibration plot was then generated using a dead-weight test sequence whereby mass was added in 1 kg increments up to a total of 20 kg and then sequentially removed, while at the same time logging the instrument displayed readings for both the load and unload sequence, Fig 6. The response indicates excellent linearity (slope of 0.998, correlation coefficient better than 0.99) with zero offset and negligible hysteresis. Similar responses were found for version three and four of the gauge. Likewise, these values were found to be stable over a three week period between initial calibration and post field trial re-testing.

### Digital Tightness Gauge: Trial 1

**Fig 7:**
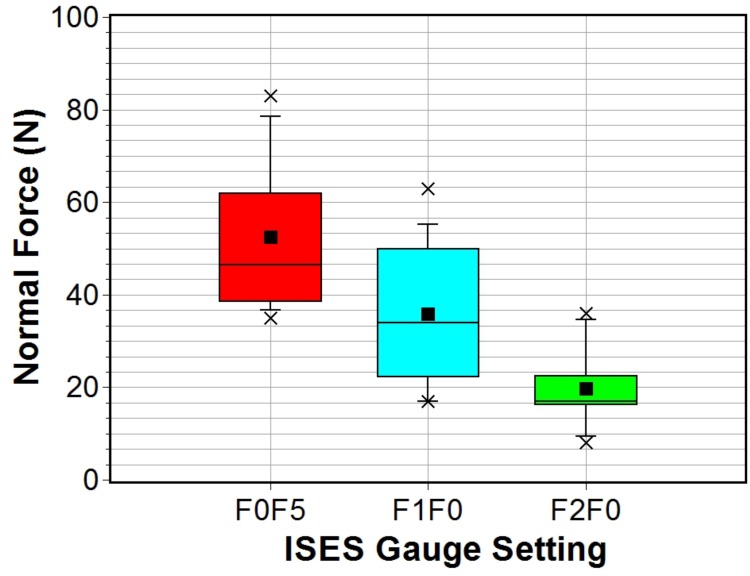
Box plot of frontal sub-noseband forces measured using DTGv3 for three ISES gauge settings. Box plot symbols for this and subsequent box-plots: solid black square—mean; rectangle lower and upper limits—25 and 75 percentiles; whiskers—5 and 95 percentiles; crosses—min and max values; line across rectangle—median.

**Fig 8:**
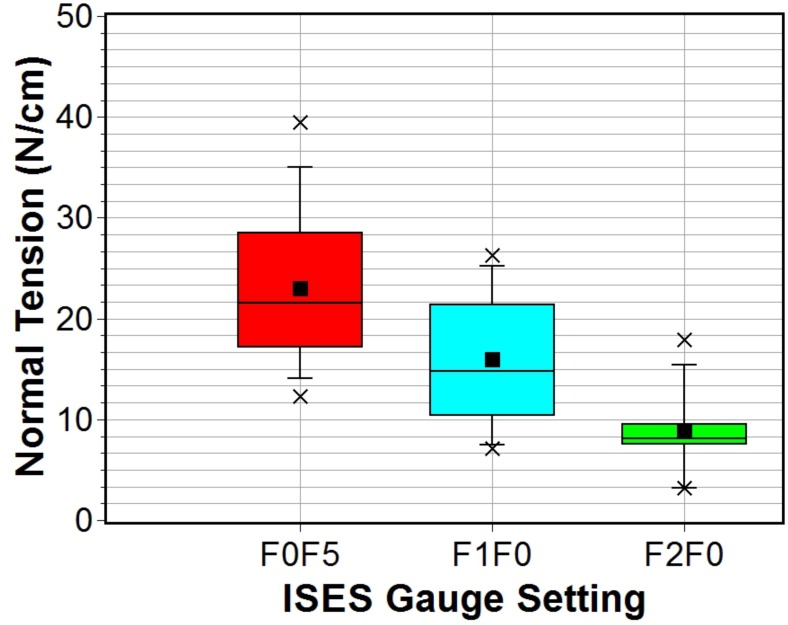
Box plot of frontal sub-noseband band normal tensions measured using DTGv3 for three ISES gauge settings.

It was possible to measure the noseband forces for all horses using DTGv3 for the frontal two (F2F0) and one finger (F1F0) tightness settings. However, some difficulty was experienced in attempts to insert the probe at the frontal site for the half finger (F0F5) setting on three horses. Therefore, the number of data points is reduced to twelve for this setting. A Shapiro-Wilk test indicated normality for all data sets at the 0.05 significance level. Descriptive statistics for DGTv3 force measurements are presented in Table 2 and the corresponding band tension statistics are presented in Table 3. Measured sub-noseband force values ranged from a minimum of 8 ± 2 N for the two finger (F2F0) setting to a maximum of 83 ± 5 N for the half finger setting (F0F5). The equivalent mass range corresponding to these forces is approximately 0.8–8.3 kg. Despite significant overlap in the range of force values measured at the different tightness band settings, there is a clear upward trend in noseband applied force (normal force) from tightness setting F2F0 to F0F5 as is clear from the combined box plot presented in Fig 7. The band tension ranged from a minimum of 3 ± 1 N/cm for the two finger setting to a maximum of 23 ± 2 N/cm for the half finger tightness setting, Table 3. The upward trend in band tension data is again clearly evident in the combined box plot shown in Fig 8.

**Table 2:** Descriptive statistics for field trial 1: DGTv3 normal force readings in Newtons (N) for different ISES taper gauge noseband settings. Sample size n.

Setting	Mean	STD	STE	Max	Min	n	Median
*F*0*F*5	52.4	16.3	4.7	83	35	12	46.5
*F*1*F*0	35.8	15.0	3.9	63	17	15	34.0
*F*2*F*0	19.8	8.1	2.1	36	8	15	17.0

**Table 3:** Descriptive statistics for field trial 1: DGTv3 derived noseband band normal tension data in N/cm, sample size n.

Setting	Mean	STD	STE	Max	Min	n	Median
*F*0*F*5	23.0	7.8	2.3	39.5	12.3	12	21.6
*F*1*F*0	15.9	6.6	1.7	26.3	7.1	15	14.8
*F*2*F*0	8.9	3.9	1.0	17.9	3.2	15	8.1

### Digital Tightness Gauge: Trial 2

**Fig 9:**
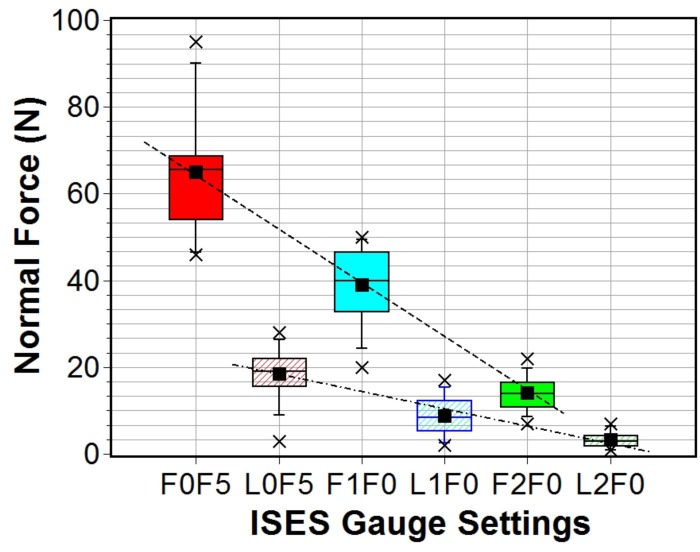
Box plot of noseband normal forces, frontal and lateral, measured using DTGv4 for three ISES gauge settings. Dotted lines added to guide the eye.

It was possible to obtain force measurements with DTGv4 on all horses for all tightness settings at the lateral site. As with trial one, readings were easily obtained for the two finger and one finger tightness settings at the frontal site. However, it was not possible to insert the gauge probe in the case of two of the horses for the half finger tightness setting at this site. Summary noseband force descriptive statistics (reflecting the reduced data set for F0F5) taken at two noseband sites using DTGv4 (trial two) are presented in Table 4. The measured force ranges from a minimum of 7 ± 1 N to 95 ± 5 N at the frontal site (equivalent to a mass range of 0.7–9.7 kg) in going from the two finger to the half finger setting. A significant reduction in overall values and range occurs for the lateral site: minimum of 1.0 ± 0.6 N for two finger tightness (L2F0) to a maximum of 28 ± 2 N for the half finger tightness (L0F5), (equivalent mass range 0.1–2.9 kg). Fig 9 shows a box plot which clearly illustrates the distinction between the values obtained at both sites and the upward trend in values in progressing from the two finger to half finger tightness for both sites.

**Table 4:** Descriptive statistics for field trial 2: DGTv4 force data in Newtons (N), sample size n.

ISES	Mean	STD	STE	Max	Min	n	Median
*F*0*F*5	65.1	15.6	4.9	95	46	10	65.5
*F*1*F*0	39.0	9.3	2.7	50	20	12	40.0
*F*2*F*0	14.2	4.1	1.2	22	7	12	14.0
*L*0*F*5	18.6	6.6	1.9	28	3	10	19.0
*L*1*F*0	8.8	4.7	1.4	17	2	12	8.5
*L*2*F*0	3.3	1.9	0.6	7	1	12	3.0

**Fig 10:**
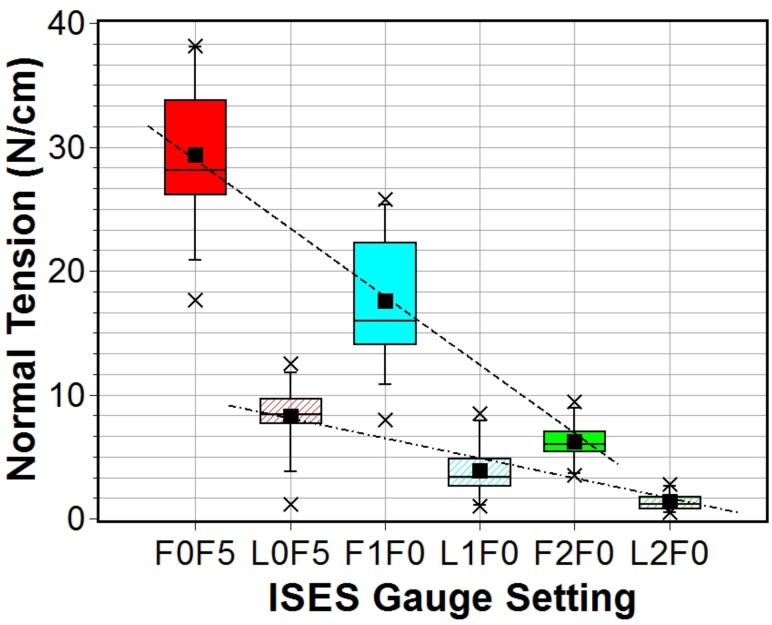
Box plot of frontal and lateral normal forces per unit width of noseband measured using DTGv4 for three ISES gauge settings. Dotted lines added to guide the eye.

Band tension data calculated from the force data (Table 4) and noseband widths were used to generate the descriptive statistics shown in Table 5. A combined box plot of the data for the three tightness settings and for both sites is shown in Fig 10.

**Table 5:** Descriptive statistics for field trial 2: DGTv4 derived band normal tension data in N/cm, sample size n.

ISES	Mean	STD	STE	Max	Min	n	Median
*F*0*F*5	29.3	6.4	2.0	38.2	17.7	10	28.1
*F*1*F*0	17.6	5.4	1.6	25.8	8.0	12	16.0
*F*2*F*0	6.2	1.7	0.5	9.5	3.5	12	6.0
*L*0*F*5	8.3	2.8	0.8	12.5	1.2	10	8.5
*L*1*F*0	3.9	2.3	0.7	8.5	1.0	12	3.3
*L*2*F*0	1.4	0.8	0.2	2.8	0.5	12	1.2

## Discussion

The force ranges measured at the frontal nasal plane (Trial 1: 8–83 N, Trial 2: 7–95 N) were similar in both trials and corresponded to noseband ISES taper gauge adjustment settings of F2F0 at the low force end and F0F5 at the high end. This range corresponds to dead weight loads in the range 1–9 kg. Noseband settings of less than F0F5 are commonly encountered at competition level [25]. It is not possible to predict what the corresponding values for zero finger tight nosebands are likely to be. A nonlinear increase in actual normal force components is likely with increased noseband tightness due to decreasing soft tissue accommodation. However, it is likely that the forces to be expected at the nasal plane with nosebands tightened to less than F0F5, i.e. taper gauge cannot be inserted at the frontal site, will be considerably in excess of the highest values measured here.

Pain in horses is difficult to measure. A range of studies into levels of force required to stimulate nociceptors in farm and laboratory animals has been reported. Cattle and sheep were found to have relatively high thresholds [26]. The mean mechanical threshold for stimulation of nociceptors on a hind limb in cattle was found to be 6.9 N in one study [27] while the corresponding force in sheep is reported as 4.9 N [28]. It is interesting to note that the device used to apply the force in the cattle study was programmed to cut out at a force value of 20 N to prevent tissue damage. It is clear from the forces measured here that noseband forces arising at localised sites on a horse’s head can be significantly larger than this cut-off threshold. The large noseband forces arising at F0F5 tightness may explain the reduced frequency of chewing, swallowing and yawning in horses with tight nosebands [12] since mouth opening will involve additional mechanical action against the noseband which will add an impulse force component or transient component to the forces already acting. Such movements have been found to produce very large peak forces in nosebands at standard recommended tightness levels [11]. It would be interesting to have dynamic data for tight nosebands, i.e. less than 1F0 ISES scale, to establish whether the use of such restrictive settings inhibit activities that could produce such transients. Generally, there is a need to investigate the pain implications of both sustained and transient contact forces on animal tissue, in particular, and animal behaviour, in general, in order to provide meaningful guidelines.

A wider noseband will result in a lower normal force per unit width for a given noseband tensile force and this will result in correspondingly lower sub-noseband pressures: the coupled force is distributed over a larger area. However, while wider nosebands may therefore reduce the risk of pain, it has been shown that blood vessel occlusion in humans occurs at lower tourniquet pressures for wide tourniquets [29]. Therefore, the effects of wider nosebands on the local circulatory system has also to be taken into account when assessing the overall physiological impact of tight nosebands. The impact of large pressure gradients such as those likely to occur along the edge of tight nosebands on the local nerves should also be considered. These gradients may be reduced to some extent by fitting foam or other cushioning materials between the noseband and the nose but the actual implications of such modifications is difficult to ascertain based on the current body of knowledge in this field.

The variability in punch hole number and spacing on individual nosebands introduced significant variability in the actual tightness setting achievable using the ISES taper gauge. Depending upon hole position the tightness of a noseband was typically set slightly looser than the setting that would have been used if noseband tightness were continuously adjustable. Ideally, head dimensions and in particular nasal plane width should be measured in order to establish an exact multiplier factor for individual animal tightness measurement. While this is possible, it was deemed impractical in the current study. However, it does point to a considerable gap in our knowledge of equine metrics and should be considered in future studies.

One horse, on which measurements were taken during trial, had a deeper than normal concave indent between the left and right nasal bones. This allowed easy insertion of the probe beneath the noseband and resulted in low force measurements at each of the three noseband settings. Such anatomical variation will not reduce the hammocking effect and may concentrate the load on the nearest convex structures (left and right nasal bone), so the registered measures will be misleading [11]. However this anatomical variation was found in one of 23 horses and is likely to occur in a small fraction of the general equine population.

Given the dimensions of the probe, elevating the noseband sufficiently to insert the probe for tightness settings less than F0F5 is not possible. However, since this tightness level is commonly used in competitions [25] there is a significant gap in our knowledge relating to magnitude of the forces and pressures likely to arise for such settings. Lower profile probes specifically tailored to such tightly fitted nosebands could be produced if such tight nosebands are deemed acceptable based on available or emerging evidence.

Current FEI guidelines stipulate that tack stewards should check each noseband for tightness at the cheek [15]. However, noseband applied force at this location is not representative of peak forces at other locations beneath the noseband and so is likely to be of limited use in estimating such peak values. In addition, variation in jaw position can radically change the geometry and hence the force/pressure applied by the noseband at such sites.

## Conclusion

The primary force acting in tightened bridle nosebands is tensile in nature. This tensile force couples to the underlying noseband support tissue where the tissue curvature is positive. Coupling does not occur where the curvature of the support tissue is negative. The value of the normal force component of the noseband tensile force is the pertinent indicator of the degree of coupling between the noseband and the support tissue in such areas of positive curvature. This is the component of the tensile force which produces compression and pressure in the underlying tissue. The normal force component of the noseband tensile force was therefore identified as an appropriate and useful practical objective measure of noseband tightness in horses involved in equine sports activities.

A digital tightness gauge capable of measuring sub-noseband normal force components was developed, calibrated and field tested. Prevailing forces will be maximal at sites of high curvature such as at the nasal bones and over the mandibular rami. The nasal plane area offers advantages over other sites for such measurements due to the clear anatomical landmarks available there and the simple and stable geometry of the site. Consequently, probe design was optimised for this site.

Normal force data measured using the digital tightness gauge for the frontal nasal plane ranged in value from 7–95 N. The corresponding normal force range for a lateral noseband site was 1–28 N. Variability of equine head metrics such as the nasal plane width and the perimeter of the nose at the noseband site introduce uncertainty in the degree to which the measured force reflects the exact force at a given site. Many of these uncertainties may be reduced or eliminated by recording actual feature dimensions such as nasal plane width for individual horses to allow use of the appropriate multiplication factor. Clearly this is not practical in a competition environment but is feasible within a research setting where accumulated data could contribute to the creation of a database of equine population metrics. Availability of such metrics could inform customized gauge design for specific population groups and categories and facilitate the development of clear differentiated noseband tightness guidelines and controls. In the absence of such data, a simple six point tightness scale is suggested. The classification scheme and break points between tightness indices will need to be informed by ongoing evidence based equine and veterinary research relating to the impact of tight nosebands on animal welfare. Successful implementation of this tightness gauge technology and its acceptance by regulatory authorities and equestrian sports bodies will be very much dependent on a productive collaborative effort between equitation scientists, veterinary researchers, and the authorities and associations with interests in equine welfare. Ultimately, the goal must be to develop a set of standards which are evidence based and informed by expert opinion and which are implementable and generally acceptable to the broad equitation community.

## Supporting Information

S1 Fig A Yato (Yato, YT-3736,) plastic profilometer used to transfer nose profile sections corresponding to normal noseband locations to paper for digitization. (a) Lateral section; (b) Transferring lateral profile to paper; (c) Frontal section; (d) Transferring frontal profile to paper.
(TIF)

S2 Fig Noseband site section profile. (a) The drawing tools in Microsoft Powerpoint 2013 were used to trace the outer profile of an imported equine head cross-section image. The traced profile was scaled to a particular adult horse dimensions, length 21.5 cm and width 14.5 cm at the noseband site. (b) The profile was digitized using the online package Webplot Digitizer http://arohatgi.info/WebPlotDigitizer/app/.
(TIF)

S3 Fig Plots of effective noseband contraction as a function of nasal plane width for different lift-off finger heights. (TIF)

S1 File MatLab Script to plot the curvature of digitized profile data. (M)

S2 File Calibration and Field Trial Data Tables (MS Excel) for DTGv3 and DTGv4. (XLSX)
