# Supplementary figures and images for: An Objective Measure of Noseband Tightness and Its Measurement Using a Novel Digital Tightness Gauge
Source: PLoS One. 2017 Jan 3;12(1):e0168996. doi: 10.1371/journal.pone.0168996 (PMC5207410; doi:10.1371/journal.pone.0168996)

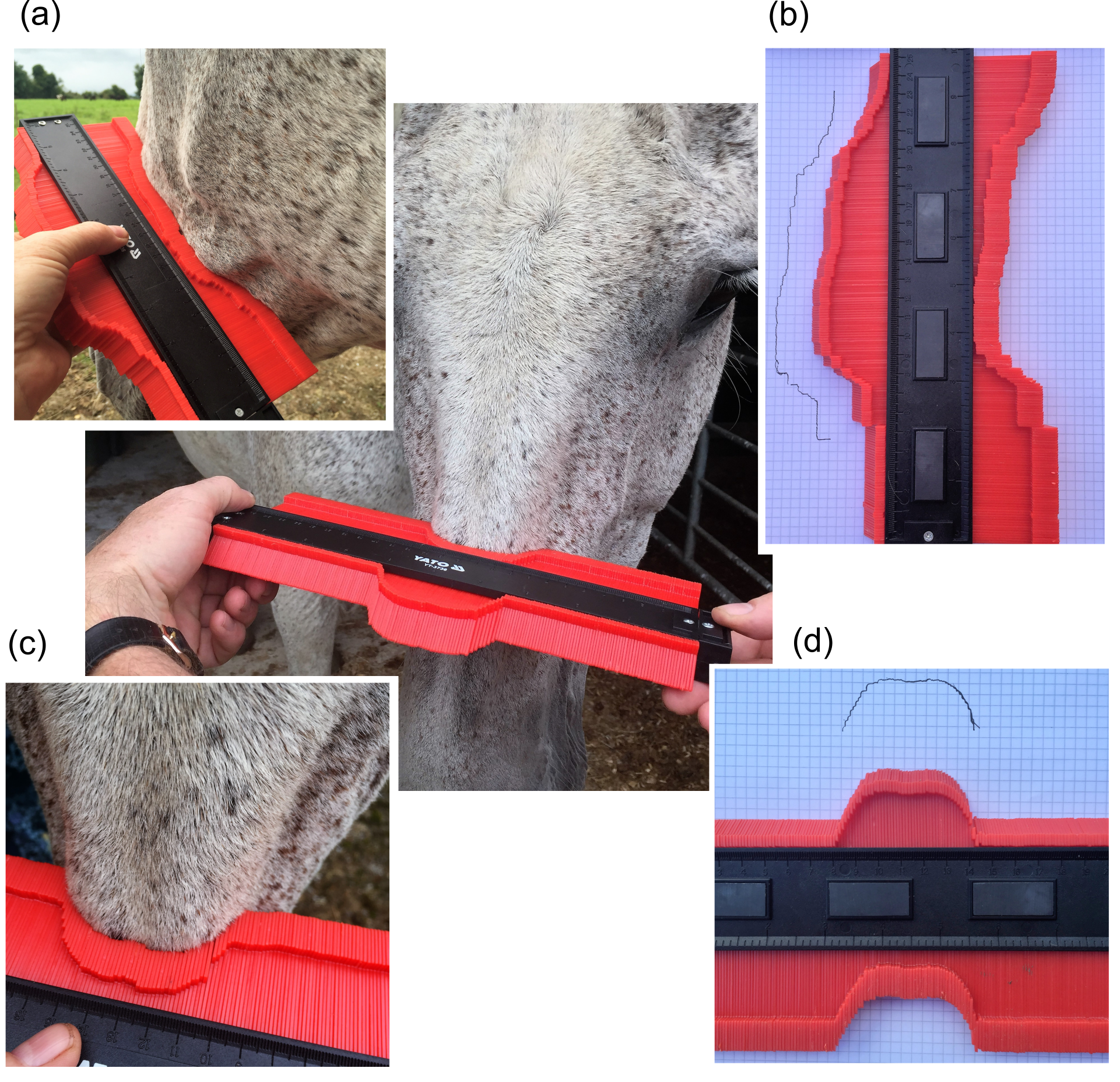

Supplement: S1 Fig — (a) Lateral section; (b) Transferring lateral profile to paper; (c) Frontal section; (d) Transferring frontal profile to paper. (TIF) [file pone.0168996.s001.tif]

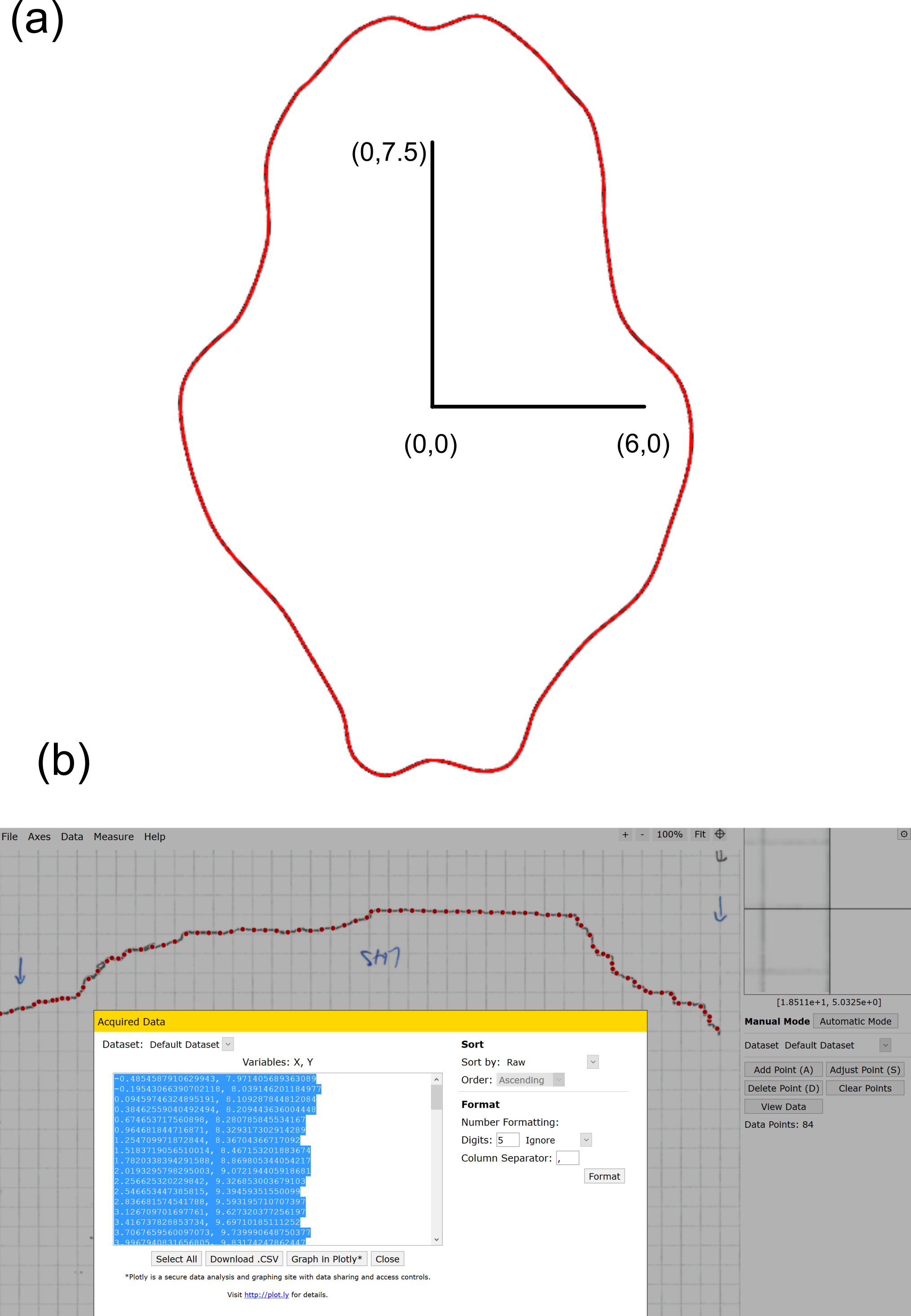

Supplement: S2 Fig — (a) The drawing tools in Microsoft Powerpoint 2013 were used to trace the outer profile of an imported equine head cross-section image. The traced profile was scaled to a particular adult horse dimensions, length 21.5 cm and width 14.5 cm at the noseband site. (b) The profile was digitized using the online package Webplot Digitizer http://arohatgi.info/WebPlotDigitizer/app/. (TIF) [file pone.0168996.s002.tif]

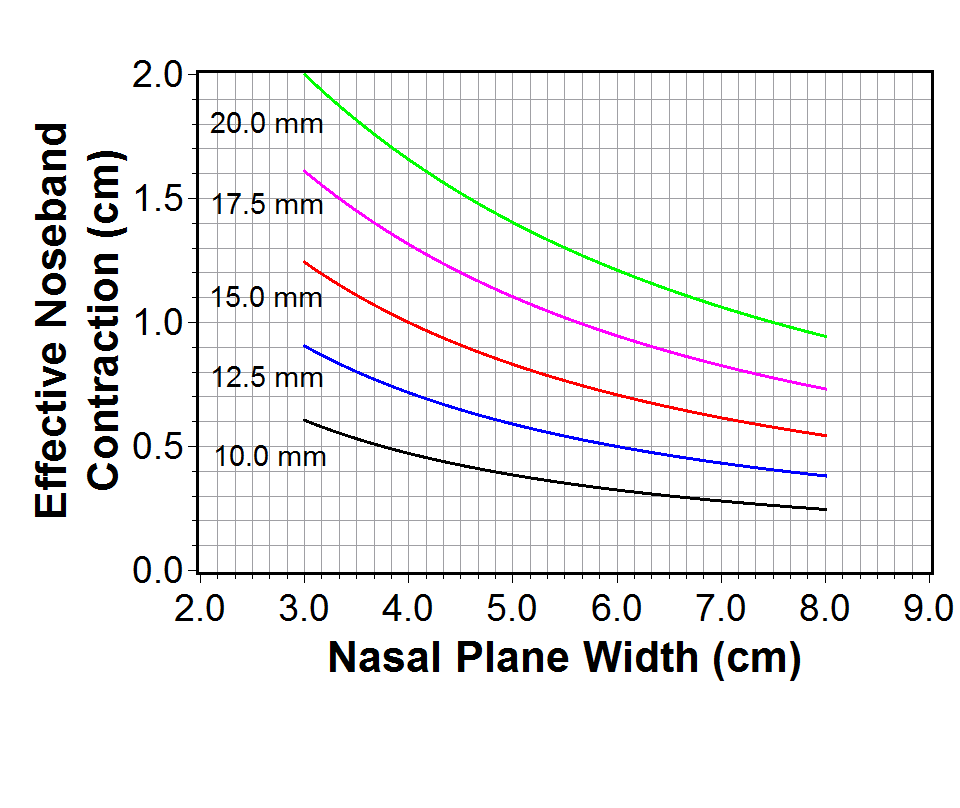

Supplement: S3 Fig — (TIF) [file pone.0168996.s003.tif]
